# Case Report: Sedation crisis caused by drug-induced sleep endoscopy in a pediatric patient with symptomatic multilevel airway obstruction

**DOI:** 10.3389/fped.2026.1825319

**Published:** 2026-05-15

**Authors:** Li'e Zeng, Jieru Lin, Yuting You, Jingyang Zheng, Fanzheng Meng

**Affiliations:** 1 Children’s Respiratory Department, Quanzhou Maternity and Children’s Hospital (Quanzhou Children’s Hospital), Quanzhou, Fujian, China; 2 Department of Pediatrics, The First Hospital of Jilin University, Changchun, Jilin, China

**Keywords:** case report, child, drug-induced sleep endoscopy, multilevel airway obstruction, sedation, tracheomalacia

## Abstract

**Background:**

Unexplained life-threatening events during pediatric sedation examinations are a major clinical challenge. Drug-induced sleep endoscopy (DISE) enables dynamic assessment of the site, severity, and pattern of airway collapse by simulating sleep through pharmacologically induced sedation. However, limited studies have utilized DISE as a diagnostic approach for elucidating the etiology of sedation-related events.

**Case presentation:**

A male patient aged 10 years was admitted with a cough for >30 days and a history of cardiopulmonary resuscitation (CPR) 2 days earlier. The patient was diagnosed with severe pneumonia at another hospital. Intravenous midazolam sedation administered during bronchoscopy preparation resulted in the development of sudden cardiorespiratory arrest. After successful CPR, the patient was transferred to Quanzhou Maternity and Children's Hospital(Quanzhou Children's Hospital). The patient experienced recurrent severe hypoxemia during sleep despite pulmonary infection control. Chest computed tomography (CT), cardiac CT angiography, echocardiography, video electroencephalography, polysomnography, and genetic testing did not reveal the underlying cause. Subsequently, the patient underwent DISE. DISE with midazolam revealed grade II tonsillar hypertrophy with posterior displacement of the tongue base restricting epiglottic elevation, which was consistent with partial upper airway obstruction. Mandibular advancement and head-and-shoulder elevation improved the obstruction. Additionally, a widened membranous portion was noted in the middle-lower segment of the trachea. The membranous portion protruded into the lumen during expiration, resulting in lumen narrowing and a reduction in lumen area by >90%, which was accompanied by blood oxygen desaturation. The final diagnosis was symptomatic multilevel airway obstruction with sedation-induced upper airway obstruction and severe dynamic tracheomalacia complicated by severe pneumonia. Home non-invasive ventilation maintained nocturnal oxygen saturation at >95% without cyanosis, bradycardia, or apnea.

**Conclusions:**

This case highlights that multilevel airway obstruction is a critical mechanism of pediatric sedation crisis. The upper airway anatomical narrowing and central airway dynamic collapse synergistically worsened under sedation. Occult tracheomalacia should be considered for sedation-related events of unclear etiology. DISE can aid in systematic airway assessment to achieve precise diagnosis, risk stratification, and safe pediatric sedation management.

## Background

Pediatric sedation is associated with safety concerns. Most adverse events of pediatric sedation are predictable and manageable. However, some children develop severe respiratory and circulatory depression that cannot be explained by conventional etiological factors, presenting diagnostic challenges. Tracheomalacia, which is characterized by excessive expiratory collapse of the tracheal wall, is a common cause of wheeze and respiratory distress in infants. The manifestations of tracheomalacia may be subtle or atypical in older children. Structural upper airway narrowing (such as tonsillar hypertrophy) may remain clinically silent until sedation-induced muscle relaxation leads to functional obstruction. The acute manifestations of these latent airway susceptibilities can be attributed to various factors, such as severe respiratory infection or sedative exposure, potentially leading to catastrophic outcomes (1).

Drug-induced sleep endoscopy (DISE) enables the comprehensive assessment of pediatric obstructive sleep apnea by directly visualizing upper airway dynamics during a sleep-like state. The diagnostic application of DISE has been expanded to other complex airway disorders as it allows dynamic, systematic evaluation from the nasopharynx to the trachea under near-physiological conditions. Thus, DISE can reveal multilevel or synergistic obstruction patterns that cannot be captured by static imaging. According to the recent international consensus, only dynamic endoscopy under sedation or natural sleep can reveal significant airway collapse and confirm occult airway malacia when awake imaging is unremarkable (2, 3). This case report reveals the application of DISE in a 10-year-old male patient with cardiorespiratory arrest during sedated bronchoscopy. In addition to confirming severe dynamic tracheomalacia, DSE identified sedation-induced upper airway obstruction, supporting a final diagnosis of symptomatic multilevel airway obstruction.

## Case presentation

### Patient information and chief complaint

A male patient aged 10 years was admitted on 17 March 2024 with a cough for >30 days and a history of cardiopulmonary resuscitation (CPR) 2 days earlier.

### History of present illness

The patient presented to a local hospital on 7 March 2024 with a cough and fever. Chest computed tomography (CT) suggested left-sided pneumonia. The patient tested positive in the *Mycoplasma pneumoniae* serological test. The symptoms did not improve after 1 week of antimicrobial therapy. On 15 March 2024, intravenous midazolam (5 mg) was administered for sedation before bronchoscopy. Approximately 2 min after midazolam administration, the child developed sudden cardiorespiratory arrest. Spontaneous respiration and circulation were restored after 7 min of CPR. Endotracheal intubation and mechanical ventilation were initiated. Subsequently, the patient was transferred to Quanzhou Maternity and Children's Hospital(Quanzhou Children's Hospital), pediatric intensive care unit (PICU) on 17 March 2024.

### Past medical history

No significant past medical history.

### Physical examination upon admission

The findings of physical examination upon admission were as follows: Body temperature: 36.5 °C; pulse: 70 bpm; respiratory rate: 25 breaths/min (mechanical ventilation); blood pressure: 117/75 mmHg; SpO₂: 95%. The patient was sedated and intubated with good ventilator synchrony. The breath sounds were coarse bilaterally and slightly diminished on the left. Cardiac or abdominal examination did not reveal aberrant findings.

### Laboratory and imaging examinations

The key laboratory findings noted on admission are summarized in Table 1.

**Table 1:** Summary of key laboratory findings on admission.

Parameter	Result	Reference range
White blood cell count	5.5 × 10⁹/L	4.3–11.3
Absolute eosinophil count	0.00 × 10⁹/L	0.00–0.68
Hemoglobin	135 g/L	118–156
Platelet count	281 × 10⁹/L	167–453
C-reactive protein	5.59 mg/L	<5
Procalcitonin	0.21 ng/mL	0–0.5
Interleukin-6	6.99 pg/mL	<5.9
Alanine aminotransferase(ALT)	39 U/L	7–30
Aspartate Aminotransferase (AST)	27 U/L	14–44
Creatinine(Cr)	28.5 umol/L	27.0–66.0
Urea	3.31 umol/L	2.7–7.0
Albumin	36.6 g/L	35–50
Lactate dehydrogenase (LDH)	244 U/L	109-245
Total IgE	35.7 IU/mL	<87
Troponin I	<0.003 ng/mL	0–0.1
B-type natriuretic peptide(BNP)	11 pg/mL	0–100

Infection and inflammation: The patient tested positive for the influenza B virus by respiratory pathogen PCR. *Mycoplasma pneumoniae* testing revealed an agglutination titer of 1:160, positive IgM (ELISA), and positive sputum by PCR. Sputum culture and smears revealed normal flora. Blood culture revealed no growth after 5 days.

Biochemistry: The coagulation profile, thyroid function, and levels of cortisol and adrenocorticotropic hormone were within normal limits.

### Imaging

Chest CT (17 March 2024) revealed pneumonia with multisegmental atelectasis of the left lung and a right tracheal bronchus. Echocardiography and cardiac CT angiography revealed no structural aberrations or vascular ring compression. Brain magnetic resonance imaging (MRI) was unremarkable. Spinal MRI suggested possible tethered cord and fatty degeneration of the filum terminale.

### Functional tests

Polysomnography (performed on April 2, 2024) revealed the following key parameters:

The apnea‒hypopnea index (AHI) was 0.1 events/hour (normal <1.5 for age), the oxygen desaturation index (ODI) was 18.31 events/hour, the minimum SpO₂ was 44%, the time with a SpO₂ < 90% was 144.7 min. The transcutaneous CO₂ monitoring was not available at our institution. These findings confirm severe sleep hypoxemia without typical obstructive sleep apnea–hypopnea syndrome, which is consistent with the diagnosis of dynamic airway collapse rather than classic OSA. A 24-hour ambulatory electrocardiogram (ECG) revealed sinus rhythm, with the lowest heart rate recorded being 55 bpm. Video electroencephalography revealed pathological changes with diffuse, irregular slow waves. Sleep cycles were difficult to determine. Pulmonary function was physiological. Genetic testing: Genetic testing was performed on June 8, 2024, using enhanced whole-exome sequencing (WES) trio (including the mother and child) and whole-genome copy number variation (CNV) analysis. Chromosomal analysis revealed that the patient's karyotype was 46,XY, with no numerical abnormalities. SNV/SV/CNV analysis: No pathogenic or likely pathogenic single-nucleotide variants (SNVs), structural variants (SVs), or copy number variants (CNVs) were identified that could explain the patient's clinical symptoms. CCHS-specific testing: Targeted testing for congenital central hypoventilation syndrome (CCHS) revealed a PHOX2B genotype of 20/20 trinucleotide repeats (normal range), confirming a negative result for CCHS. In summary, no evidence was found to support a genetic etiology for the patient's airway obstruction or sedation-related cardiorespiratory events.

### Clinical course and diagnostic dilemmas

After admission, the patient received mechanical ventilation and anti-infective therapy. Two bronchoscopies with bronchoalveolar lavage were performed during mechanical ventilation (17 March and 20 March 2024). Follow-up chest CT (24 March 2024) revealed partial absorption of pulmonary lesions. After weaning from mechanical ventilation, the patient developed recurrent severe hypoxemia during sleep. The SpO₂ and heart rate decreased to 80% and 38 bpm, respectively. The patient's parents reported that during natural sleep, he exhibited loud snoring, irregular breathing patterns, and recurrent episodes of perioral cyanosis. The cyanosis resolved spontaneously upon repositioning (side-lying) or awakening. These observations were consistent with the polysomnography findings of severe sleep hypoxemia without typical obstructive sleep apnea. On 12 April 2024, bronchoscopy was attempted without endotracheal intubation. After intravenous midazolam (5 mg) administration, the child rapidly developed cyanosis and hypoxemia. The SpO₂ and heart rate decreased to 85% and 48 bpm. Consequently, the procedure was terminated. Despite a comprehensive evaluation, the etiology of sedation/sleep-induced life-threatening events was unclear.

### Definitive diagnostic technique: DISE (procedure and findings)

DISE was performed on 5 May 2024 to determine the etiology and observe airway dynamics in a highly controlled environment.

Setting and safety monitoring: DISE was performed in the PICU with a senior pediatric respiratory endoscopist, a senior PICU physician trained in pediatric advanced life support, and an anesthesiologist. Sedation was achieved through slow intravenous infusion of midazolam, targeting a sleep depth of ≥ stage II to minimize the suppression of upper airway muscle tone and respiration and maintain a bispectral index of 50–60 (4). The ECG parameters, blood pressure (non-invasive), SpO₂ levels, and end-tidal CO₂ levels were continuously monitored. After the target sleep depth was achieved, a flexible bronchoscope (VisionScopic IPX7 ENF-X20, F2410009; outer diameter 3.1 mm) was transnasally introduced for systematic evaluation.

Rationale for sedation with midazolam during DISE: We acknowledge that reusing midazolam in a patient with prior midazolam-induced cardiorespiratory arrest required careful risk-benefit assessment. The decision was based on the following considerations: dose and titration: A slow intravenous infusion was used (starting at 0.05 mg/kg/h, titrated to effect), rather than the previous rapid bolus (5 mg given as a single intravenous push). The target was light-to-moderate sedation (bispectral index 50–60), not deep sedation. Alternative agents considered included dexmedetomidine; however, its longer half-life and potential for bradycardia/hypotension were concerns given the patient's baseline bradycardic episodes. In accordance with the Chinese expert consensus on drug-induced sleep endoscopy (2025 edition) (4) and international DISE protocols (5), midazolam is an established sedative option for DISE. The adverse events during the initial procedure were attributed to rapid bolus administration and the patient's underlying airway pathology, not an idiosyncratic drug reaction. Risk mitigation: The procedure was performed in the PICU with a multidisciplinary team, continuous monitoring, and immediate availability of resuscitation equipment. After receiving a detailed explanation of the risks, the patient's legal guardian provided written informed consent.

### Findings

Immediately after achieving sedated sleep (bispectral index 50–60), the patient gradually developed audible snoring that intensified over time. On chest auscultation, inspiratory stridor and expiratory wheezing were noted. Mild subcostal and intercostal retractions were observed. No cyanosis, tachypnea, bradypnea, or respiratory pauses were noted.

**Figure 1:**
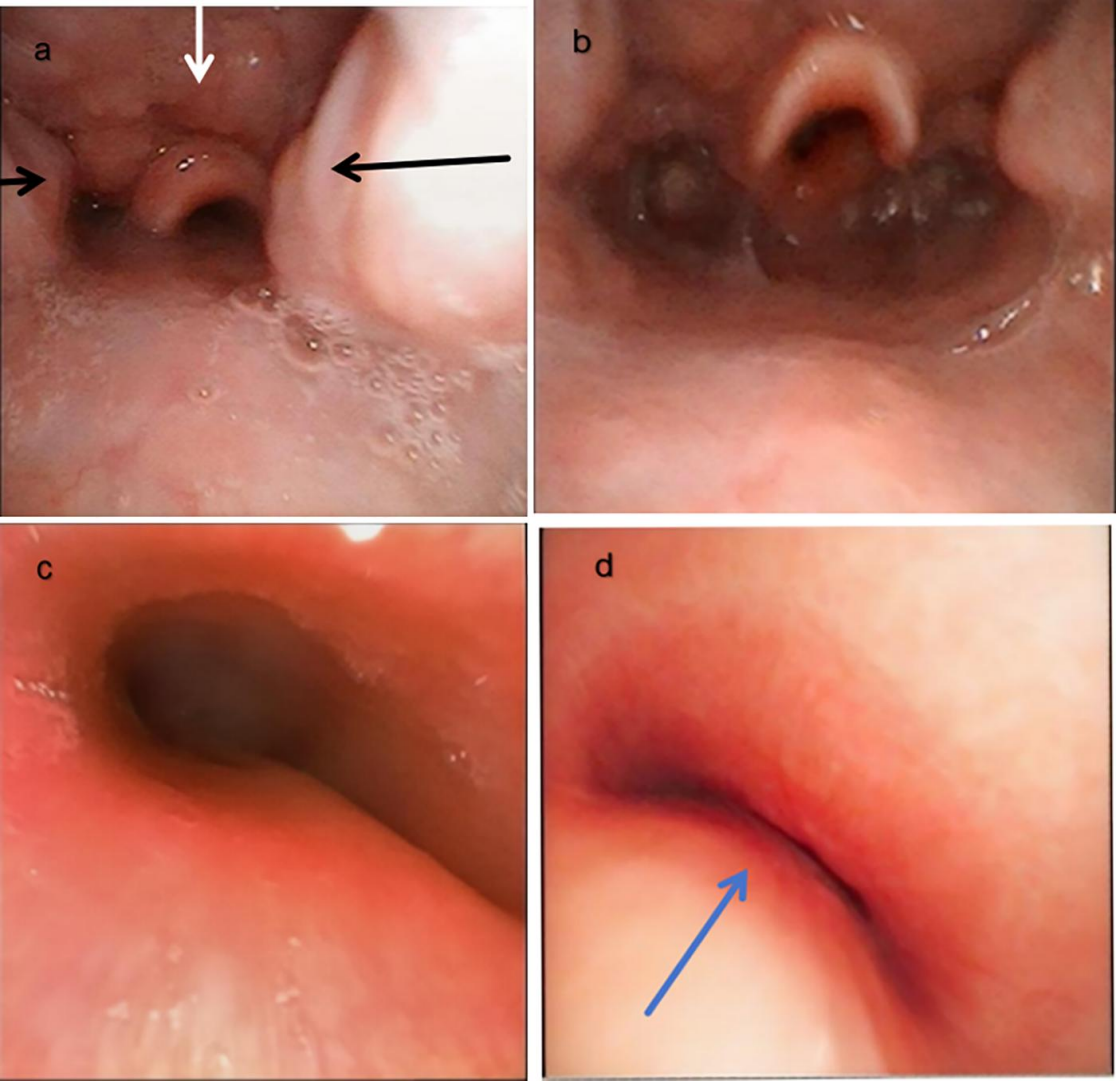
Drug-induced sleep endoscopy (DISE) findings. **(a)** Upper airway obstruction under sedation: bilateral grade II tonsillar hypertrophy (black arrow) and tongue base compression of the epiglottis (white arrow). **(b)** The obstruction improved with mandibular advancement and head-and-shoulder elevation. **(c)** Partial tracheal collapse during expiration: the tracheal membrane bulges anteriorly, reducing the lumen diameter by 50%–60%. **(d)** Severe dynamic tracheal collapse during expiration: lumen area reduction >90% (blue arrow).

A systematic evaluation was performed from the nasopharynx to the main bronchi. Velum (soft palate) level: The anterior-posterior diameter was mildly reduced, but concentric lateral wall collapse was not observed. Hypopharynx: The lateral walls did not exhibit excessive dynamic collapse toward the midline during inspiration. Upper airway obstruction was primarily localized to the oropharyngeal level (bilateral grade II tonsillar hypertrophy) and the tongue base-epiglottic level (posterior displacement of the tongue base compressing the epiglottis), which is consistent with partial upper airway obstruction (Figure 1a). Mandibular advancement with head-and-shoulder elevation immediately improved airway patency, as evidenced by the resolution of audible snoring, disappearance of inspiratory stridor, and a reduction in chest wall retractions (Figure 1b). Central airway: A wide membranous portion was observed in the middle-lower segment of the trachea. The membranous portion gradually protruded into the lumen during expiration, narrowing the lumen narrowing and decreasing the lumen area by >90%. These changes were accompanied by oxygen desaturation (Figures 1c,d). Other central airways: The main bronchi and intermediate bronchus were also systematically examined during DISE. No significant dynamic collapse was observed in the left or right main bronchi or in the intermediate bronchus. Airway patency in these segments was maintained throughout the respiratory cycle.

Physiological association: The tracheal collapse occurred synchronously with progressive SpO_2_ decline. The SpO_2_ rapidly returned to physiological levels after the patient returned to the awake state. This mimicked the pathophysiological process of sedation crisis and provided decisive functional evidence.

### Outcome and follow-up

The final diagnosis was symptomatic multilevel airway obstruction (sedation-induced upper airway obstruction with severe dynamic tracheomalacia) and severe pneumonia. After discharge, the child received long-term home non-invasive ventilation. Nocturnal SpO₂ was maintained in the physiological range during ventilator use. The SpO₂ levels during daytime nap were in the range of 85%–94% without ventilator support. The key clinical events, diagnostic tests, and management decisions are summarized in Table 2.

**Table 2:** Timeline of key events, examinations, and management.

Time point	Clinical manifestations and events	Key checks and findings	Diagnosis and intervention
2024-03-07	Cough and fever onset	Chest CT from external hospital: Left pneumonia; *Mycoplasma pneumoniae* IgM positive.	Diagnosis: Community-acquired pneumonia
2024-03-15	First sedation crisis: Sudden respiratory and cardiac arrest occurred during bronchoscopy under midazolam sedation.	Bronchoscopy not completed; ROSC after CPR.	Admitted to the PICU of our hospital, with tracheal intubation and mechanical ventilation.
March 17,2024 to early April	Pneumonia resolved, but recurrent severe hypoxemia.. during sleep after extubation	Polysomnography (04-02): Severe sleep hypoxemia (atypical OSA). No etiology on cardiac CTA, brain/spinal MRI, or genetic tests	Dilemma: Etiology unclear. Suspect occult dynamic airway obstruction
2024-04-12	Second sedation crisis: Cyanosis and hypoxemia immediately after midazolam; procedure aborted.	Clinical evidence confirms: the crisis is directly related to the sedative drug (midazolam).	Key clue: Clearly, indicates sedation-induced dynamic airway obstruction, providing strong indication for DISE examination.
2024-05-05	Drug-induced sleep endoscopy (DISE) was performed under close monitoring.	Upper airway: Grade II tonsillar hypertrophy and tongue base collapse → partial obstruction, improved by jaw-thrust. Central airway: Expiratory tracheal membrane prolapse (mid-to-lower trachea) → > 90% luminal reduction with desaturation.	Diagnosis: Symptomatic multilevel airway obstruction (Sedative-induced upper airway obstruction + severe dynamic tracheomalacia).
After discharge (long-term management)	Nocturnal SpO₂ normal with NIV; daytime nap SpO₂ 85%-94% without support.	Home non-invasive ventilation (CPAP mode) assisted ventilation.	Management: Long-term NIV as “pneumatic stent”; patient flagged as high-risk for sedation.

ROSC, return of spontaneous circulation; DISE, drug-induced sleep endoscopy; PICU, Pediatric intensive care unit; OSA, Obstructive sleep apnea; CPAP, Continuous positive airway pressure; NIV, Non-invasive ventilation; CPR, Cardiopulmonary resuscitation.

## Discussion and conclusions

This case study reports a pediatric case of respiratory and cardiac arrest after midazolam sedation. The patient, who was transferred to our hospital, continued to experience recurrent severe hypoxemia during sleep despite pulmonary infection control. Hypoxemia occurred primarily during sleep or sedation. Awake oxygen levels and routine tests did not explain the recurrent desaturation with bradycardia. DISE performed under near-pathogenic conditions revealed a multilevel mechanism in a single session. In particular, the patient was diagnosed with partial upper airway obstruction and severe dynamic tracheomalacia. DISE also provided therapeutic guidance through positional responsiveness, highlighting its value in complex pediatric airway disorders.

### Differential diagnosis

Given the severity of the clinical course (cardiorespiratory arrest, recurrent hypoxemia, bradycardia), we systematically evaluated and excluded the following conditions:

Eosinophilic or allergic lung diseases: The total IgE (35.7 IU/mL) and absolute eosinophil count (0.01 × 10⁹/L) were within normal ranges. No clinical features of asthma or allergic bronchopulmonary aspergillosis were present.

Vasculitis or systemic inflammatory conditions: Inflammatory markers (CRP 5.59 mg/L, PCT 0.21 ng/mL) were only mildly elevated, which is consistent with the resolution of pneumonia. No clinical signs of vasculitis (rash, arthritis, renal involvement) were observed.

Neuromuscular disorders affecting airway tone: Genetic testing for congenital hypoventilation syndromes was normal. Physical examination revealed no hypotonia, weakness, or bulbar dysfunction.

Central hypoventilation syndromes: Polysomnography did not reveal the characteristic pattern of central hypoventilation. The patient had a normal awake respiratory drive and maintained adequate ventilation during wakefulness without clinical evidence of chronic hypoventilation (e.g., no daytime somnolence, morning headache, or cyanosis). Additionally, genetic testing for PHOX2B mutations was negative.

Other causes of dynamic airway collapse: Cardiac CTA excluded vascular rings and external compression. The DISE findings (dynamic collapse limited to the trachea with a widened membranous portion) were most consistent with primary tracheomalacia rather than compression from mediastinal masses or vascular anomalies.

### Mechanism of sedation crisis: progression of a marginally stable airway toward decompensation

Anesthetic agents used for DISE may induce airway obstruction that does not manifest during natural sleep (6). In this patient, grade II tonsillar hypertrophy and tongue base collapse constituted an anatomical substrate that increased inspiratory resistance. Midazolam may exacerbate these manifestations by reducing airway muscle tone (7). Upper airway obstruction induces forceful inspiration, increasing intrathoracic negative pressure (8). This negative pressure further amplifies central airway collapse and worsens tracheal narrowing (9). In the study patient, the mucosal portion of the middle and lower trachea protruded into the lumen, obstructing more than 90% of the lumen, which was the direct result of this mechanical magnification. The endoscopic findings in this patient—dynamic >90% expiratory reduction of the tracheal lumen with a wide, posteriorly protruding membranous portion—are consistent with the concept of expiratory central airway collapse (ECAC). ECAC encompasses both excessive dynamic airway collapse (EDAC, where the cartilage is intact) and tracheomalacia (where the cartilage is structurally weakened). DISE suggests primary tracheomalacia, while the marked worsening under sedation highlights the dynamic nature of the obstruction. Future studies using adjunctive measures such as endobronchial optical coherence tomography may help differentiate between these entities. Therefore, post-sedation cardiorespiratory arrest is consistent with sedation-induced multilevel airway decompensation. This suggests that children with sleep-related symptoms or unexplained sleep hypoxemia should be managed using high-risk sedation and airway protocols.

### Diagnostic value of DISE for pediatric sedation-related events

This case highlights the limitations of conventional tools for assessing dynamic airway obstruction (10). Static examinations (such as chest CT and cardiovascular imaging) and awake bronchoscopy may fail to capture aberrations that manifest only during sleep/sedation. Polysomnography can quantify hypoxic burden but often cannot localize obstruction, define morphology, or relate collapse to movement/position, contributing to diagnostic uncertainty after repeated life-threatening events. DISE can reproduce and visualize airway changes during sedation-sleep states in a controlled and safe environment (11). In this study, DISE confirmed severe dynamic tracheomalacia and upper airway collapse. This dynamic visualization connects occult structural abnormalities to clinical events (12, 13).

### Clinical implications and future directions

Symptomatic tracheomalacia cases may be overlooked, especially in older children without typical wheeze/stridor. Tracheomalacia may remain clinically silent until major stressors (such as severe pneumonia or sedatives) induce decompensation (14). Previous studies have reported that the manifestations may vary depending on the age and that sedation or severe infection may serve as triggering factors (15). The reasons for the manifestation of severe symptoms only after pneumonia are unclear. We propose a dual-effects hypothesis: acute inflammation and edema reduce airway reserve, promoting decompensation under sedation (16). Severe inflammation may also cause persistent airway instability via structural remodeling or neurogenic pathways (17, 18). This can potentially explain the ongoing need for respiratory support during sleep even after clinical recovery. This hypothesis links infection–inflammation–airway remodeling with dynamic airway disease and suggests proactive dynamic airway assessment in children with a history of severe respiratory infection (19).

DISE enabled the identification of an extremely high-risk status for future sedation/anesthesia and provided a mechanism-based justification for long-term non-invasive ventilation as a “pneumatic stent” (20). This diagnostic strategy considers early DISE or other dynamic airway assessments in patients with persistent sleep-related hypoxemia after resolution of acute infection, any unexplained sedation-related events, or suspected multilevel obstruction despite non-diagnostic static imaging (2, 3, 21). Based on the sedation sensitivity of these patients, DISE should be performed with strict safety measures, including precise sedation titration, continuous monitoring (SpO₂, ECG, and end-tidal CO₂), readiness for difficult airway management, and deployment of an experienced resuscitation-capable team.

### Strengths and limitations of this study

This case highlights the diagnostic value of DISE in identifying occult multilevel airway obstruction and demonstrates its safe application in a high-risk child. Limitations include the inherent generalizability constraints of a single case report. Awake bronchoscopy was not performed in this patient, as the procedure was considered potentially risky given the history of a sedation-induced life-threatening event and was unlikely to reveal the dynamic collapse that manifested only under sedation. Therefore, a direct comparison between awake and sedated findings is not available. The follow-up duration was short, and long-term outcomes remain to be determined. Subtle genetic or neuromuscular factors have not been exhaustively explored. Despite these limitations, this case provides clinically significant insights.

### Patient perspective

We never imagined that a simple cough could turn into such a nightmare. When our son stopped breathing during the bronchoscopy, we thought we were going to lose him. After transfer to this hospital, he kept having episodes of oxygen drops during sleep, and we were afraid to let him fall asleep.

When the doctors suggested another sleep endoscopy (DISE), we were scared. However, they explained everything carefully and assured us that they would be extra careful. Watching the procedure on the screen, we finally understood why he kept turning blue—his airway was collapsing like a soft straw. Now, with the breathing machine at home, he sleeps peacefully through the night. We are so grateful to the team who never gave up.

The parents provided written informed consent for the publication of this case report.

## Conclusions

In this patient, upper airway anatomical narrowing and central airway dynamic collapse synergistically worsened under sedation, leading to cardiorespiratory arrest. This case suggests that multilevel airway obstruction may underlie sedation-related events in susceptible children. DISE proved valuable in identifying this underlying pathology. Our findings are hypothesis-generating rather than definitive. Future prospective studies are needed to determine the prevalence of occult airway malacia among children with unexplained sedation-related events. Based on this experience, we suggest that clinicians consider dynamic airway assessment (including DISE where available) when conventional evaluations fail to explain severe, recurrent sedation-induced respiratory events.

## Data Availability

The raw data supporting the conclusions of this article will be made available by the authors, without undue reservation.
