# Supplementary material for: Case Report: Sedation crisis caused by drug-induced sleep endoscopy in a pediatric patient with symptomatic multilevel airway obstruction
Source: Front Pediatr. 2026 May 15;14:1825319. doi: 10.3389/fped.2026.1825319 (PMC13219248; doi:10.3389/fped.2026.1825319)
Supplement: Supplementary file 1 [file Table1.docx]

### ****CARE Checklist (2013) for:Case Report: Sedation crisis caused by drug-induced sleep endoscopy in a pediatric patient with symptomatic multilevel airway obstruction****

| ****Item No**** | ****Item Description**** | ****Page/Line No.**** | ****Brief Explanation (if needed)**** |
| --- | --- | --- | --- |
| ****1**** | The diagnosis or intervention of primary focus followed by the words "case report" | Title page | Title: Case Report: Sedation crisis caused by drug-induced sleep endoscopy in a pediatric patient with symptomatic multilevel airway obstruction |
| ****2**** | 2 to 5 key words that identify diagnoses or interventions in this case report, including "case report" | Abstract / Keywords section | Keywords: drug-induced sleep endoscopy, tracheomalacia, child, sedation, multilevel airway obstruction, case report (6 keywords, including "case report") |
| ****3a**** | Introduction: What is unique about this case and what does it add to the scientific literature? | Introduction section | Background section: Describes the diagnostic challenge of unexplained sedation-related crises and the emerging role of DISE. |
| ****3b**** | Main symptoms and/or important clinical findings | Abstract / Case presentation | Abstract/Case presentation: "cough for >30 days", "sudden cardiorespiratory arrest after midazolam", "recurrent severe hypoxemia during sleep". |
| ****3c**** | The main diagnoses, therapeutic interventions, and outcomes | Abstract / Case presentation | Final diagnosis: symptomatic multilevel airway obstruction (sedation-induced upper airway obstruction + severe dynamic tracheomalacia). Intervention: home non-invasive ventilation. Outcome: nocturnal SpO₂ >95%. |
| ****3d**** | Conclusion—What is the main "take-away" lesson(s) from this case? | Abstract / Conclusions | Highlights that multilevel airway obstruction is a critical mechanism of pediatric sedation crises, and DISE is essential for systematic airway assessment. |
| ****4**** | One or two paragraphs summarizing why this case is unique (may include references) | Introduction section | Describes rarity of using DISE to diagnose sedation-related crises and concept of multilevel obstruction |
| ****5a**** | De-identified patient specific information | Case presentation, Patient information | "A male patient aged 10 years" – age and sex only, fully anonymized. |
| ****5b**** | Primary concerns and symptoms of the patient | Case presentation, History of present illness | Cough, fever, sudden cardiorespiratory arrest after sedation. |
| ****5c**** | Medical, family, and psycho-social history including relevant genetic information | Case presentation, Past medical history; Functional tests | "No significant past medical history". Genetic testing: WES trio, PHOX2B normal |
| ****5d**** | Relevant past interventions with outcomes | Case presentation, History of present illness | Bronchoscopy attempt at outside hospital resulted in cardiorespiratory arrest. |
| ****6**** | Describe significant physical examination (PE) and important clinical findings | Case presentation, Physical examination | Vital signs, breath sounds coarse bilaterally, slightly diminished on left. |
| ****7**** | Historical and current information from this episode of care organized as a timeline | Table 1 | Timeline of key events, examinations, and management. |
| ****8a**** | Diagnostic testing (such as PE, laboratory testing, imaging, surveys) | Case presentation, Laboratory/imaging/Functional tests | Full list of tests performed. |
| ****8b**** | Diagnostic challenges (such as access to testing, financial, or cultural) | N/A | Not applicable – no such challenges encountered. |
| ****8c**** | Diagnosis (including other diagnoses considered) | Clinical course and diagnostic dilemmas; Outcome and follow-up | Describes exclusion of common etiologies; final diagnosis in Outcome |
| ****8d**** | Prognosis (such as staging in oncology) where applicable | N/A | Not applicable. |
| ****9a**** | Types of therapeutic intervention (such as pharmacologic, surgical, preventive, self-care) | Outcome and follow-up | Long-term home non-invasive ventilation. |
| ****9b**** | Administration of therapeutic intervention (such as dosage, strength, duration) | Outcome and follow-up; Table 1 | Described as "long-term home non-invasive ventilation"; CPAP mode implied (detailed in Table 1). |
| ****9c**** | Changes in therapeutic intervention (with rationale) | N/A | No changes – treatment was initiated and maintained. |
| ****10a**** | Clinician and patient-assessed outcomes (if available) | Outcome and follow-up; Patient perspective | Clinician: nocturnal SpO₂ normal with NIV. Patient perspective: parent-assessed outcomes |
| ****10b**** | Important follow-up diagnostic and other test results | Outcome and follow-up | SpO₂ during daytime naps 85%-94% without NIV. |
| ****10c**** | Intervention adherence and tolerability (How was this assessed?) | Patient perspective | Parent reports "he sleeps peacefully through the night for the first time" – implies good adherence and tolerability. |
| ****10d**** | Adverse and unanticipated events | Clinical course and diagnostic dilemmas; Findings | Two sedation-related crises (3/15 and 4/12). No adverse events reported during DISE or NIV use. |
| ****11a**** | A scientific discussion of the strengths AND limitations associated with this case report | Discussion, Strengths and limitations of this study | Dedicated section discussing both strengths and limitations |
| ****11b**** | Discussion of the relevant medical literature with references | Entire Discussion section | Extensively cites relevant literature on DISE, tracheomalacia, sedation mechanisms. |
| ****11c**** | The scientific rationale for any conclusions (including assessment of possible causes) | Discussion, Mechanism of sedation crisis; Diagnostic value of DISE | Provides mechanistic explanation for the findings |
| ****11d**** | The primary "take-away" lessons of this case report (without references) in a one paragraph conclusion | Conclusions section | Summarizes key lessons without references. |
| ****12**** | The patient should share their perspective in one to two paragraphs on the treatment(s) they received | Patient perspective section | Parent describes their experience and gratitude |
| ****13**** | Did the patient give informed consent? Please provide if requested | Declarations, Consent for publication | Written informed consent for publication... was obtained from the patient's parent/legal guardian. |
